# Space-for-Time Substitution Works in Everglades Ecological Forecasting Models

**DOI:** 10.1371/journal.pone.0081025

**Published:** 2013-11-21

**Authors:** Amanda I. Banet, Joel C. Trexler

**Affiliations:** Department of Biological Sciences, Florida International University, Miami, Florida, United States of America; Institut Maurice-Lamontagne, Canada

## Abstract

Space-for-time substitution is often used in predictive models because long-term time-series data are not available. Critics of this method suggest factors other than the target driver may affect ecosystem response and could vary spatially, producing misleading results. Monitoring data from the Florida Everglades were used to test whether spatial data can be substituted for temporal data in forecasting models. Spatial models that predicted bluefin killifish (*Lucania goodei*) population response to a drying event performed comparably and sometimes better than temporal models. Models worked best when results were not extrapolated beyond the range of variation encompassed by the original dataset. These results were compared to other studies to determine whether ecosystem features influence whether space-for-time substitution is feasible. Taken in the context of other studies, these results suggest space-for-time substitution may work best in ecosystems with low beta-diversity, high connectivity between sites, and small lag in organismal response to the driver variable.

## Introduction

Ecological forecasting uses scientific data to model how environmental scenarios will affect future ecosystems, ecosystem services, and natural capital [1]. Carefully applied models are valuable in environmental management because they allow decisions to be based on the best available science, and improve communication between scientists and managers. However, the predictive power of these models depends on the quantity and quality of the data used to determine the statistical relationship between an environmental driver and the ecosystem response. The generally agreed upon convention is that this relationship is best determined by looking at replicated study sites over the course of an extended period of time [2]. However, in many cases long-term time-series data are not available. As an alternative, researchers sometimes substitute spatial data for temporal data in their models, with the assumption that the spatial relationship between the environmental driver and the response variable can be used as a proxy for the temporal relationship. Collecting data with large spatial coverage over a short period of time allows researchers to increase the range and quantity of data points used to determine the relationship between an environmental driver and the response of the ecological variable of interest without the constraint of waiting for many years of data to be collected. 

This approach has been used widely across fields. For example, the study of succession has extensively employed space-for-time substitution (often called chronosequence in this field) to quantify long-term vegetation change [3,4]. More recently, climate change studies have used space-for-time substitution (bioclimatic envelope models) to examine how predicted changes in climate will affect species range and distribution [5–7]. It is also regularly used to guide decisions for ecosystem management [8,9]. Models that use space-for-time substitution have the potential to be an extremely useful tool for ecosystem management because often, people have not had the means or foresight to collect long-term data for species or ecosystems that are now deemed to have high ecological or cultural value.

However, not all researchers have accepted space-for-time substitution as a valid way to create predictive models. A proposed problem of this method is that factors other than the target driver could be affecting ecosystem response, and these factors may vary spatially [10]. This could produce a misleading correlation between the target variable and ecosystem response, though the degree to which this is a problem is not agreed upon in the literature [11–15]. Disagreement between studies is likely a function of several factors, including temporal and spatial scaling of models/predictions, and community composition and processes [16,17].

In the Florida Everglades, restoration efforts have focused on the recovery of wading bird breeding populations to historical levels [18]. Because nesting success of wading birds is tied to the presence of food sources such as small fishes and crustaceans [19,20], a considerable amount of work has focused on the relationship between environmental drivers that have been controlled by managers and engineers for much of the last century (e.g. hydrology) and populations of these animals. Recent Everglades forecasting models have used long term monitoring data from the Modified Water Deliveries Project (Mod Waters) to determine the relationship between hydrology and density of small fishes and crustaceans [21]. In more recent years, the Comprehensive Everglades Restoration Plan (CERP) has included a monitoring program that collects similar data over a larger spatial scale. These data give us a remarkable opportunity to explore whether spatial data can be a valid substation for temporal data in ecological forecasting models used for ecosystem management. Here we use temporal and spatial monitoring data from the Florida Everglades to examine how scaling affects the performance of spatial-substitution models that predict bluefin killifish (*Lucania goodei*) population response to a drying event. We then discuss our results in the context of other published studies to determine if certain ecosystem features are indicators of whether space-for-time substitution is a viable alternative to long-term studies.

## Methods

Effects of spatial and temporal scale of data on forecasting ability were explored using coefficient of determination (r^2^) and bias values as indicators of model performance. Models of varying temporal and spatial extent were created using two empirical datasets from ongoing monitoring programs in the Everglades, USA, a karstic wetland with seasonal drying. The first dataset, called Mod Waters, is from a long-term monitoring program and is both temporally and spatially rich. The second dataset, collected as part of the Comprehensive Everglades Restoration Plan (CERP), covers a larger spatial area than Mod Waters but has fewer years of data and fewer samples per year. Sample collection methods are identical between these two studies and are described in Trexler et al. [21]. We first use bootstrapped replicates of the Mod Waters data to examine how varying spatial and temporal extent affects a model’s ability to predict another dataset. We then use bootstrapped replicates of the CERP data to examine how increasing spatial extent beyond what is included in the Mod Waters dataset affects the predictive ability of the models.

### Datasets

Mod Waters data used in this study is based on 18,935 throw trap observations taken from June 1996 to May 2008 in the Florida Everglades. Throw traps are enclosure traps that quickly encompass a well-defined area, and provide an accurate representation of fish density, size, and community structure [22]. Sampling efforts covered 3,125.95 km^2^ and 12 complete wateryears. A wateryear spans from June until May and encompasses an entire dry (approximately June through November) and wet (approximately December through May) season; e.g. Wateryear 1997 runs from June 1996 through May 1997.

**Figure 1:**
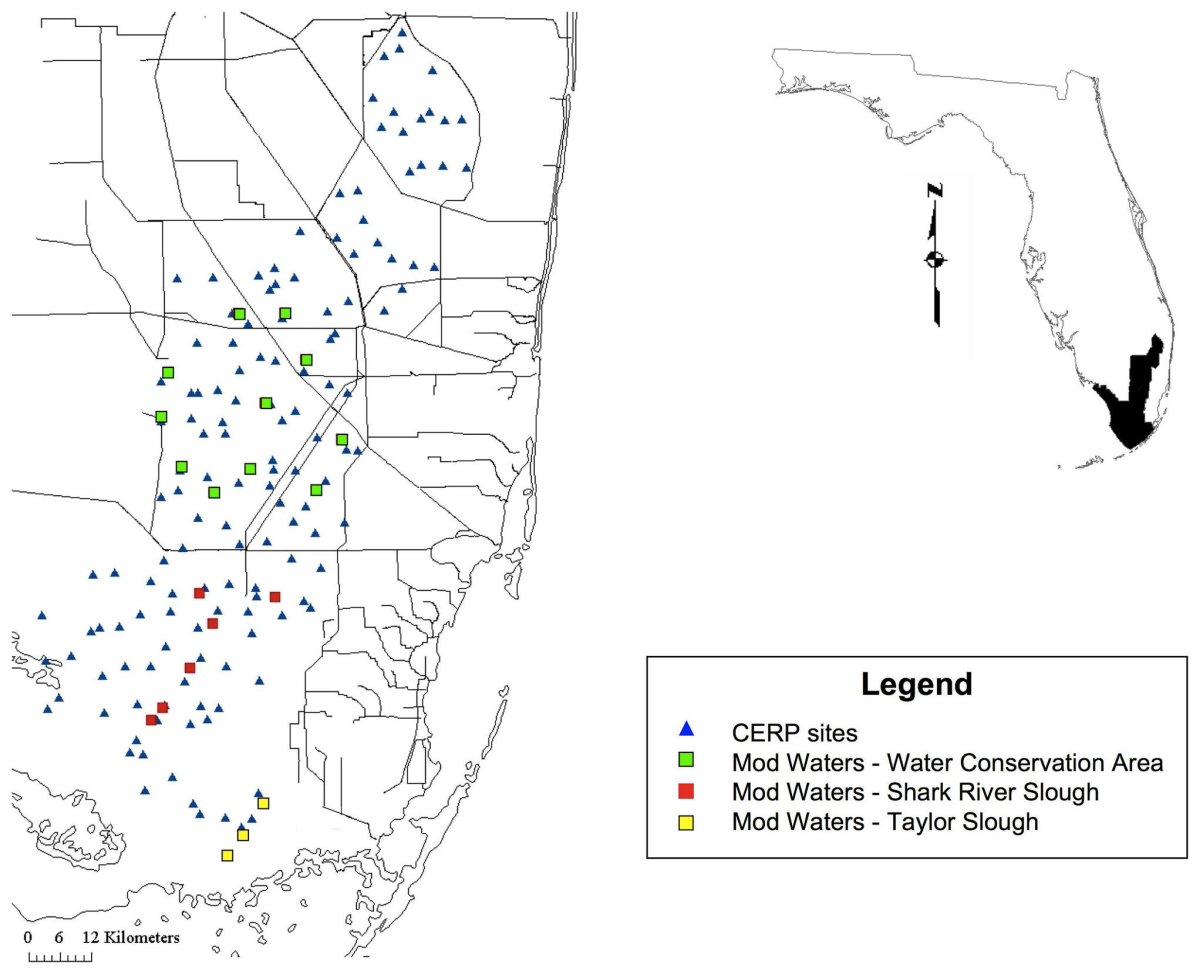
Mod Waters and CERP sampling sites in the Florida Everglades. CERP models were based on three years of data pooled from all sampling sites. Mod Waters models were broken down into three, six, nine, and twelve years of data. Spatial scales used were Ecosystem (all Mod Waters sites), Regions (sites within Water Conservation Area, Shark River Slough, or Taylor slough), individual sites, and plots (not shown – 100 m2 areas within each site).

For the purposes of this study, we considered the ecosystem to be the entire sampling area, including three regions (Figure 1: Taylor Slough (TSL, 218.27 km^2^), Shark River Slough (SRS, 788.97 km^2^), and Water Conservation Areas (WCA, 2,118.71 km^2^). Nested within each region are sites (three in TSL, six in SRS, and eleven in WCA). Sites are 1 km^2^. Within each site are three to five plots. Plots cover approximately 100 m^2^. 

The CERP monitoring program began in 2005, and covers 11 regions in addition to those included in Mod Waters, making up approximately 50,902 km^2^. CERP uses a generalized random tessellation stratified (GRTS) sampling design in order to produce a more spatially balanced sample (Stevens and Olsen 2004). CERP predictive models included the entire spatial coverage area and were based on three years of contiguous data (2006-2008) in order facilitate direct comparison with Mod Waters results.

### Model Creation and Parameterization (Overview in Figure 2A)

**Figure 2:**
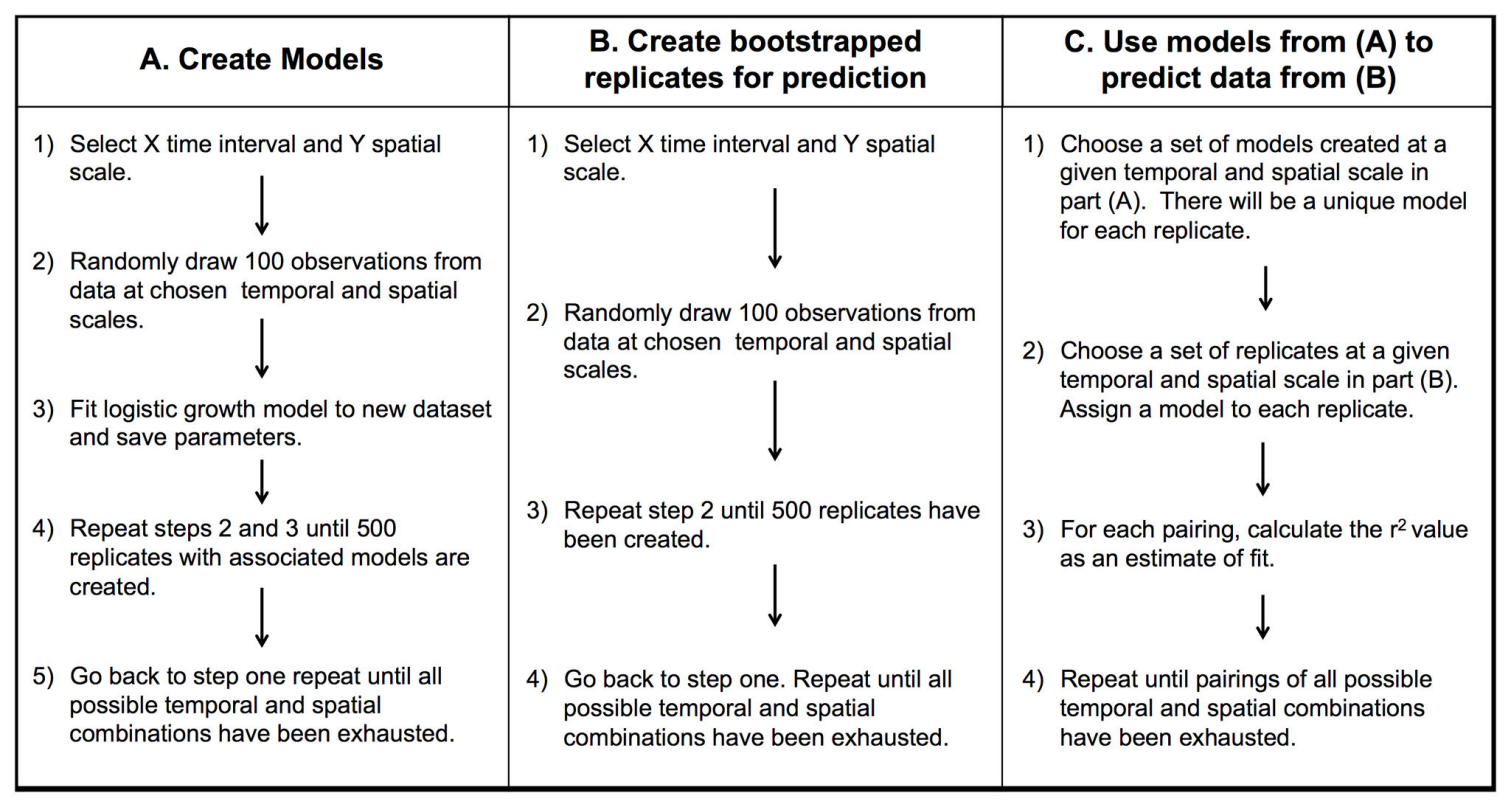
Overview of methods. Flow chart providing an overview of a) model creation, b) creation of datasets for prediction, and c) application of models to datasets for prediction. Note that the details for each data set differs. Details for step (a) can be found under the heading *Model* *Creation* and *Parameterization* in the methods section. Details for steps (b) and (c) are available under the heading *Model* *Application* in the methods section.

In Mod Waters analyses, temporal scale was broken into four groups: three, six, nine, and twelve years. Spatial scale was also classified into four groups, based on the sampling program design described above. In order of increasing size, these were plots, sites, regions, and ecosystem. This classification of the data allowed us to create a set of models that differ in the range of temporal and spatial data (e.g. models could include data spanning a large temporal scale and a short spatial scale, a small temporal scale and a large spatial scale, small temporal and spatial scales, or large temporal and spatial scales, and so forth). CERP data added an additional, larger scale and used only three years of data. Thus, models from this dataset included our largest spatial scale and shortest temporal scale.

We chose to use a population-level analysis for this exercise, which is relevant for species-specific management scenarios or when individual species serve as a performance metric for ecosystem health, as in the Florida Everglades. In choosing an appropriate forecasting model for this study, we looked for a species that was abundant and amenable to the creation of predictive models using temporal data. Using a species with these characteristics is more likely to reveal the usefulness of each approach because it eliminates poor performance due to small sample size (as opposed to temporal and spatial scaling issues). Bluefin killifish (*Lucania goodei*) are abundant in the Florida Everglades and have been deemed an indicator species because of their importance to wading birds. Logistic growth models based on plot means from the Mod Waters dataset have been shown to be a good descriptor of the relationship between species density and the number of days since the habitat last re-flooded following drying using long-term monitoring data [23]. Because logistic grown models that predict bluefin killifish population density in response to the number of days since an area was last dry (DSD) have recently been used to inform management decisions [24], we chose to use the same logistic growth models in this exercise in order to make the exercise relevant to current management practices, as follows:

$$Log\left(FISH+1\right)=\frac{K}{\left(1+{\left(\frac{\left(K-Y_{0}\right)}{Y_{0}}\right)}^{\left(-r*DSD\right)}\right)}$$

where *DSD* = days since last dry down, *r* = growth constant, *FISH* = total Bluefin killifish density (number of individuals) per meter, *K*=asymptotic density, and Y_*0*_ = Y-intercept

Creating a set of models directly from the collected data would provide indicators of model fit, but would not provide information about the underlying distribution of that value, such as a measure of the standard error. In contrast, fitting models to a set of bootstrapped datasets provides an indirect method to calculate the properties of the underlying distribution [25]. Thus, simple bootstrapping with replacement was used to create 500 replicates with 100 observations for each combination of spatial and temporal scales of the Mod Waters dataset, described above. The sampling unit was data collected from a single throw-trap collection. Using this approach, spatial extent (rather than grain) was increased as spatial scale increased, as it expanded the area from which plot means could be sampled. 

It is important to note that the models created from the original (not bootstrapped) dataset would typically contain many more than 100 observations and as a result have higher r^2^ values than the models in our exercise. We chose to cap the number of observations at 100 for two reasons: 1) creating equal sample sizes for each replicate allowed us to disentangle the r^2^ values from the number of observations, and 2) because our main interest was to use the relative values of r^2^ to compare the performance of different models (rather than actual value of r^2^) capping the observations reduced the time required to do the bootstraps to a manageable timeframe. 

Because data from smaller spatial scales encompass more spatial levels – e.g. there is only one ecosystem but many plots – and there were 500 replicates for each category, the smaller spatial scales ended up with a comparatively larger number of data sets. However, repeated tests showed that 500 replicates stabilized r^2^ values within 0.02 units at all levels, indicating that this difference will not strongly affect the final results.

Two types of bootstrapped samples were used for model creation from Mod Waters data in order to explore how correlation based on temporal closeness affects forecasting results. We created “contiguous year” models using chronological years, beginning in 1997 (e.g. three-year models were based on 1997-1999, six-year models based on 1997-2002, and so on). Non-contiguous models randomly chose the years that were included in each replicate (e.g. three year models could contain data from years 1997, 2002 and 2004, or any other random sample of three years). Logistic growth models were fit to each space/time combination and parameters from each model were then used to predict the log bluefin killifish density at all other space/time combinations. 

CERP predictive models included the entire spatial coverage area and were limited to three years of contiguous data in order make these results comparable to the Mod Waters results. Model creation followed the same bootstrapping methods described for the Mod Waters data. 

### Model Application (Overview in Figure 2B and C)

Parameters from the models created above were used to predict bluefin killifish density in another set of bootstrapped datasets created from the Mod Waters dataset, henceforth referred to as the datasets for prediction. Spatial scale of the datasets for prediction was classified into the same four groups as the datasets used for model creation: ecosystem, regions, sites, and plots. Temporal scale was broken into four groups as well, but these groups differed from the datasets used for model creation. In the datasets for prediction, all temporal groups included data spanning three contiguous year groups: 1997-1999, 2000-2002, 2003-2005, or 2006-2008. This allowed us to examine how well our model developed from three years of Mod Waters data (1997-1999) could predict bootstrapped datasets from the same three years, and groups of three years that were progressively farther in the future. This second set of bootstrapped data also contained 500 replicates with 100 observations for each combination of spatial and temporal scales. 

To help clarify how the models were applied, consider the following example. If we were interested in how a model created from a large spatial scale, but small temporal scale (e.g. a model created from data over the whole ecosystem but only included data from 1997-1999), predicted a dataset from a smaller spatial scale at different time period (e.g. bootstrap replicates created from data at the site level and included years 2006-2008), each observation in a replicate of the dataset to be predicted would be paired with a randomly chosen model created from one of the 500 replicates at the ecosystem spatial scale and 3-year (1997-1999) temporal scale. Though specific spatial and temporal scales were used in this example, this strategy could be used regardless of the temporal scale of the model or the predicted dataset. Comparison of the predicted and observed bluefin killifish density allows a mean r^2^ value to be calculated, which provides information on how well a model created at a given spatial and temporal scale can predict another dataset. Additionally, because we bootstrapped the datasets, we are able to calculate a standard error for each r^2^ value. 

Profile analysis [26] was used to determine the effect of temporal and spatial scale on r^2^ values. A one-way ANOVA was used to test for differences in model fit between year groups in the dataset that was predicted. For all analyses, we checked that statistical assumptions were met. Bias in our estimator of model fit (systematic deviation of our predicted model from the observed data) was calculated by subtracting the observed log bluefin killifish density from the predicted value. Non-linear models such as the logistic growth model used in this exercise may be particularly susceptible to bias when the shape of the data does not match the chosen model. In order to help us determine whether the models had similar bias for the range of the predictor variable, bins were created to examine bias at varying days since dry (0-250, 251-500, 501-750, 750-1000, and >1000 DSD). This allowed us to easily pull out trends from the large amount of data.

## Results

### Model Fit

#### Fit of original models

The fit of Mod Waters models to the data they were created from is shown in figures S1A and S2A (online supplement). Note that these values are lower than what is typically found in Florida Everglades management scenarios because we limited our sample size to 100 observations per replicate. The r^2^ value of the original CERP model was 0.26.

#### Predictive ability of contiguous & non-contiguous year models

**Figure 3:**
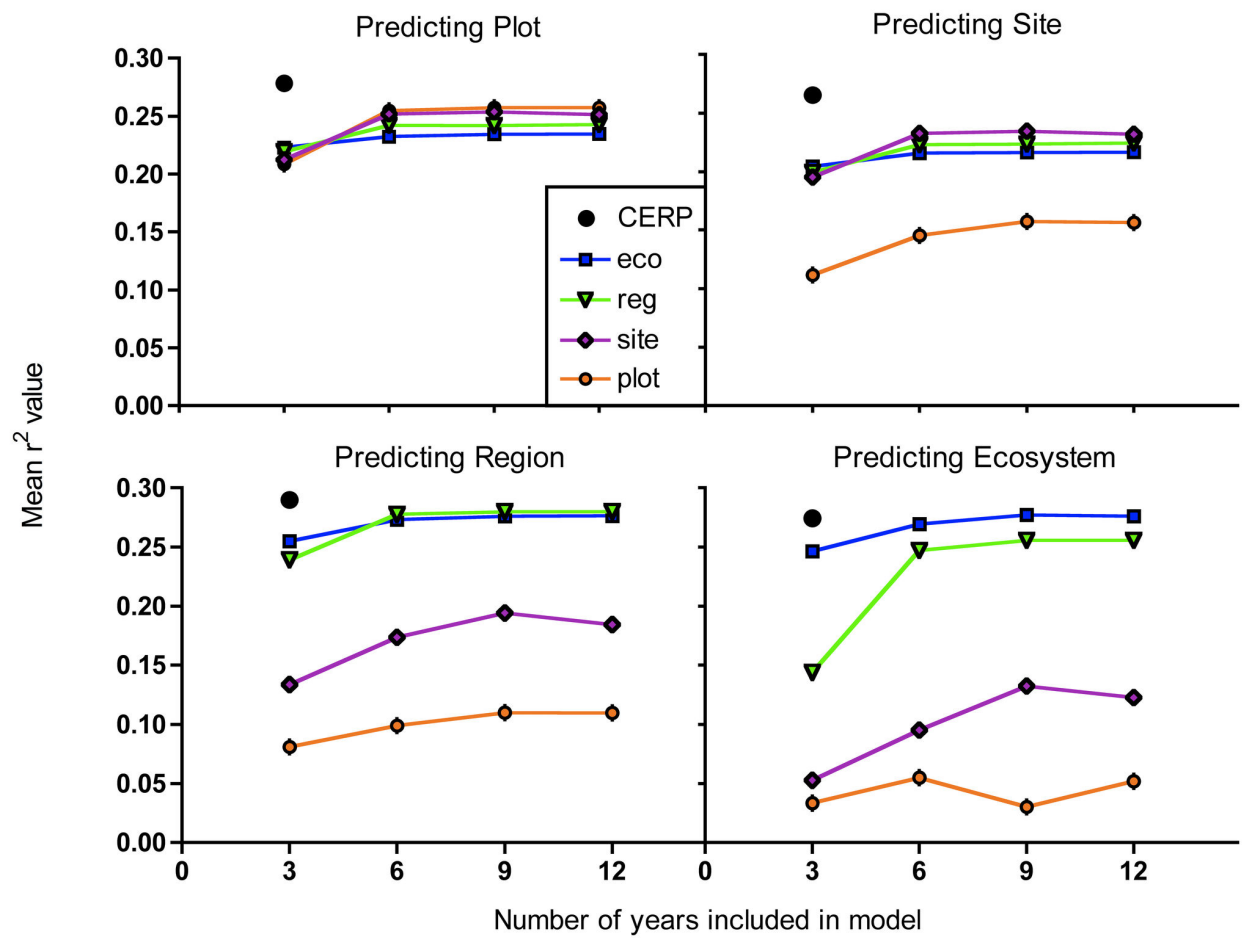
Mean r^2^ values of contiguous year models when predicting different spatial scales. Values are averaged across all groups of years that were predicted in order to make trends easier to extract visually. The corresponding non-contiguous figures can be found on the online supplement. Error bars are present, but are so small they are not visible. Details of the unpooled groups follow the same trends and can be seen in figures S1 and S2.

We pooled r^2^ values for all year groups (in the predicted data set) in order to see how time, space, and the interaction between the two affected model fit when predicting bluefin killifish density at different spatial extents. Models created from both contiguous (sequential) and non-contiguous (randomly selected) years showed the same general patterns. Time, space, and the interaction between the two were all significant factors in determining model fit (Table 1, Figure 3, Figure S3). Though all factors were significant, examination of the F-values in Table 1 allows us to parse out the relative contribution of each. Increasing both temporal and spatial scale increased the r^2^ value of the models, but as the spatial scale being predicted became larger, the importance of temporal scale decreased (yet was still highly significant). Conversely, when smaller spatial scales were being predicted, increasing spatial scale had a smaller, yet still highly significant, influence. The interaction was more important when predicting smaller spatial scales: Increasing temporal scale had a larger positive influence on the r^2^ value when the spatial scale the models were based on was small. However, as the spatial scale being predicted became larger, the importance of the interaction was reduced and spatial scale included in the model dominated (Table 1, Figure 3, Figure S3). A closer examination of these trends, which parses out the four temporal-year groups predicted by the data, rather than pooling them as in table 1 and figures 3 and S3, is available in figures S1B and S2B.

**Table 1:** F and p-values of profile analysis for contiguous and non-contiguous year models.

	**Spatial Scale Being Predicted**			
	**Plot**	**Site**	**Region**	**Ecosystem**
**Contiguous years**				
Years	F_3, 1.01e_^6^ = 16448.00	F_3, 334023_ = 5626.11	F_3, 71565_ = 1907.45	F_3, 23037_ = 1559.24
	p<0.0001	p<0.0001	p<0.0001	p<0.0001
Space	F_3, 336367_ = 63.95	F_3, 111341_ = 1232.30	F_3, 23855_ = 2062.78	F_3, 7679_ = 3182.93
	p<0.0001	p<0.0001	p<0.0001	p<0.0001
Interaction	F_9, 1.01e_^6^ = 1199.94	F_9, 334023_ = 365.03	F_9, 71565_ = 95.4	F_9, 23037_ =301.76
	p<0.0001	p<0.0001	p<0.0001	p<0.0001
**Non-contiguous years**				
Years	F_3, 976269_ = 5061.51	F_3, 334326_ = 2394.39	F_3, 65574_ = 904.03	F_3, 23865_ = 734.36
	p<0.0001	p<0.0001	p<0.0001	p<0.0001
Space	F_3, 325423_ = 186.05	F_3, 111442_ = 1821.51	F_3, 21858_ = 2612.97	F_3, 7955_ = 3720.50
	p<0.0001	p<0.0001	p<0.0001	p<0.0001
Interaction	F_9, 976269_ = 1304.34	F_9, 334326_ = 597.91	F_9, 65574_ = 242.55	F_9, 23865_ = 206.97
	p<0.0001	p<0.0001	p<0.0001	p<0.0001

Analysis of Contiguous and Non-contiguous years were performed separately. Independent variables included the spatial and temporal scales which original model was created. The dependent variables were the coefficient of determination when the models were predicting data at the plot, site, region, and ecosystem levels. All factors were significant. Examination of the F-values give information regarding the relative importance of time and space in models when predicting different spatial scales.

**Figure 4:**
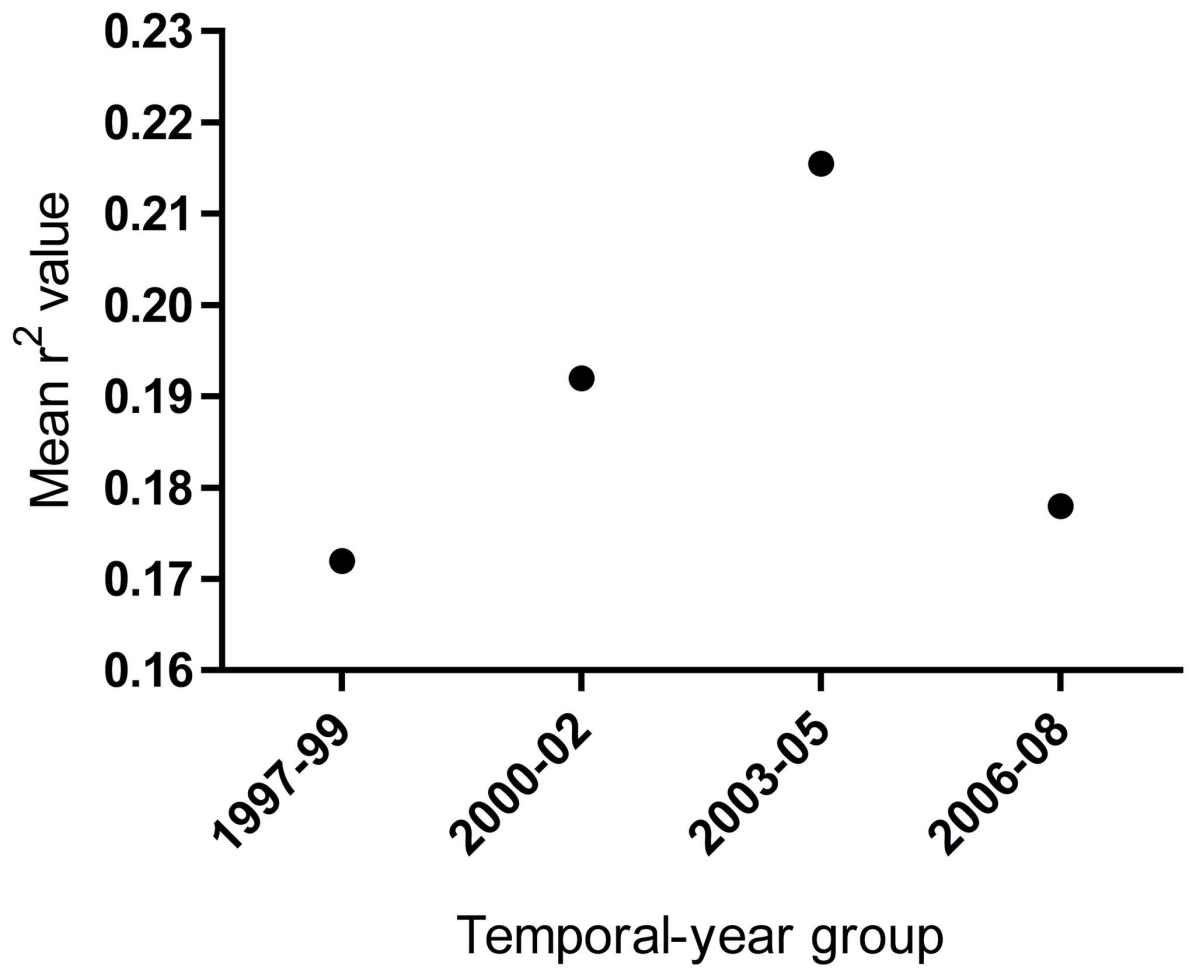
Mean r^2^ values of contiguous three-year Mod Waters model predictions. Models based on data from 1997-99 were used to predict data from various temporal-year groups (the same three years, the following three years, and so on). This allowed us to determine whether the models’ ability to predict other data was consistent even as the data being predicted became farther removed temporally from the data the model was created on. Error bars are present, but are so small they are not visible. Though year groups differed significantly, this appears to be due to factors other than temporal distance from the time period that was used to create the model, as it is not a linear trend.

Models based on three years of Mod Waters data (1997-99) were used to predict bootstrapped replicates from 1997-99, 2000-02, 2003-05, and 2006-08. This allowed us to determine whether model performance decreased when used to predict data farther into the future. Though year-groups differed significantly in r^2^ values (One-way ANOVA: F_3,528832_ = 1335.49, p<0.0001), there was not a decreasing trend in value as the temporal distance between the data the model was created on and the data it was predicting increased (Figure 4).

#### Predictive ability of CERP models

CERP models were based on contiguous years from 2006-2008. Non-contiguous models were not created since it is a temporally smaller dataset. CERP models created with only three years of data performed better than or equal to Mod Waters models in all instances (Figure 3, Figure S4). 

### Bias

#### Contiguous & non-contiguous year models

Bias (systematic deviation of our predicted model from the observed data) was calculated by subtracting the observed log bluefin killifish density from the predicted value. Most models exhibited relatively small bias, though both contiguous and non-contiguous year models exhibited large spikes for certain bins (Tables S1 & S2). Further investigation showed that these spikes were the result of a very small percentage of the total number of models (fewer than 1% had a bias greater than 5 in each bin) that severely overestimated the population density. The large bias values occurred when a model was created on data that spanned a smaller range of the dependent variable than the dataset it was predicting. In contiguous year models, this problem was most apparent when small spatial scales were predicting large spatial scales. Increasing temporal range of the models did not alleviate the problem (Table S1). In non-contiguous year models, certain year combinations produced a larger number of inferior models than the contiguous year models. For these models, increasing the number of years included reduced the occurrence of spikes in bias values (Table S2).

#### CERP models

All CERP models included a sufficiently large range of DSD values and thus did not exhibit the spikes in bias seen in some of the Mod Waters models (Table S1).

## Discussion

Our results suggest that in the Florida Everglades, space-for-time substitution in predictive models not only works, it can produce predictions commensurate with models created from temporal data. This is in contrast to a number of other studies [2,4,13,14,17,27]. Our results suggest that while increasing both spatial and temporal scale does improve model fit, managers with limited resources or those that need to make management decisions without having a long history of monitoring data may be able to use spatial-for-time substitution in their models without sacrificing accuracy of their model’s predictions if care is taken to include adequate spatial coverage in the samples. Clearly, we cannot extend our results to all ecosystems. However, these results do tell us that the current paradigm, that models created from spatial data cannot match the predictive ability of those created from temporal data, is flawed. The question is not whether space-for-time substitution is valid: instead, we ask under what circumstances can it provide commensurate predictive ability? 

One contributing factor may be the degree of temporal lag of the target variable in response to the predictor variable(s), which can manifest itself differently depending on the data from which the models are created. Lauenroth and Sala [28] compare temporal and spatial model performance when predicting annual net primary productivity (ANPP) of a shortgrass steppe in north-central Colorado. They found that regression models created using spatial rainfall and ANPP data had a steeper slope than those created using temporal data. Another study found similar results in the Konza Prairie LTER (long term ecological research) site [29]. Lauenroth and Sala suggest the difference of results between methods in their study arose from a temporal lag in the time required for vegetation to capitalize on the amount of precipitation available at a given time. A snapshot of conditions such as that used when creating spatial models will not reveal how rainfall in the more distant past has shaped current vegetation. As explained in Kratz et al. [2], vegetation is a composite of the average conditions of the site, so that even in dry years, historically wet sites will exhibit vegetation consistent with a wetter site. In our study, bluefin killifish have a small lag time, quickly repopulating an area in response to the influx of water. Additionally, the predictor variable used in our study was DSD. By nature, this variable includes information about the past and may assist in accounting for error associated with time lags. This is supported by previous attempts at predicting bluefin killifish densities that used single-dimensional predictor variables such as water depth; the models produced did not perform as well (Trexler, unpublished data). Combined, these results provide insight into one possible way spatial models may be improved, particularly when the relationship being explored is related to climatic factors such as precipitation. For example, in younger monitoring programs information on species distribution and density may be temporally limited. However, data on climate variables is often available for a longer time span, having been collected for different purposes. An a priori expectation that a lag is present (based on the biology of the organism) could be incorporated into models. Lags of varying lengths can be explored in order to find the best fit.

Another potential influence on the validity of the space-for-time trade-off is the relative roles that dispersal and local adaptation in community composition. High dispersal rates can cancel out the selective pressures that may be present with abiotic differences between sites, making biota more homogenous across space. Conversely, communities with lower dispersal are more likely to exhibit increased among-site diversity (also called beta-diversity) [30,31]. Sites that are well connected promote dispersal that reduces beta-diversity [32] and reduced spatial variation has been shown to create better models when substituting space for time [15]. Wetlands such as the Florida Everglades have high interconnectedness between sites and dispersal can shape spatio-temporal patterns of fish populations [33]. The European Commission Communication on the wise use and conservation of wetlands argues that “Wetlands should not be considered in isolation but as forming a global interconnecting network, often between distant areas” and asserts that wetlands serve as a path for the colonization of new habitats and genetic exchange [34]. Bluefin killifish populations from this study demonstrate this point. Genetic analysis shows spatial homogeneity across the Everglades for two fish taxa [35,36]. A recent review of the use of chronosequences in the study of succession [4] points out that space-for-time substitution produced erroneous predictions in many classic cases of succession study with the exception of wetlands. 

The spikes in bias demonstrated in some temporal and spatial combinations in this study convey a fundamental caveat regardless of the type of data being used: Using models to predict data outside the range from which it was created is dangerous, particularly when the shape of the relationship between the driver and target variables is not well understood. When the shape of this relationship is understood, models can sometimes cautiously be extrapolated beyond their original range. For example, in this study the relationship between DSD and bluefin killifish density is best described by a logistic growth model, which plateaus at some time scale determined by the model parameters. If the original models include a large enough range of data that we can confidently define where this plateau occurs, extrapolating beyond that should produce reasonable predictions. However, if the data used to create the model have not reached a plateau, the predictions are likely to be unsatisfactory. In general, non-linear relationships are susceptible to this problem.

We have shown that the traditional idea that temporal data is always better than spatial data for creating predictive models is not always true. We have also proposed two potential characteristics of an ecosystem that will help determine whether space-for-time substitutions are valid: lag in organismal response to predictor variables and the degree of beta-diversity across spatial sites. While the studies presented here are consistent with these ideas, further investigation is required to determine the generality of these claims. Connectivity and low beta-diversity, as exhibited in wetlands, may be necessary factors in determining whether space-for-time substitutions are effective, but there may also be other characteristics of wetlands that make them good candidates. We encourage future studies to focus on simulation models that incorporate different levels of connectivity and beta-diversity in order to address this claim. Likewise, models that incorporate different levels of lag in organism response to predictor variables can provide insight on what types of variables will provide the best descriptions of population dynamics when spatial substitutions are made.

## Supporting Information

Figure S1 **Heat map of contiguous year model fits.** A) “Heat map” representation of fit for models created from a) contiguous years from the Mod Waters Data and b) The models from (a) fit to all combinations of groups of three contiguous years and spatial scales.
(TIFF)

Figure S2 **Heat map of non-contiguous year model fits.** A) “Heat map” representation of fit for models created from a) non-contiguous years from the Mod Waters Data and b) The models from (a) fit to all combinations of groups of three contiguous years and spatial scales.
(TIFF)

Figure S3 **Mean r^2^ values of non-contiguous year models when predicting different spatial scales.** Values are averaged across all groups of years that were predicted. Error bars are present, but are so small they are not visible.
(TIFF)

Figure S4 **Mean r^2^ values when models from CERP data predict Mod Waters data.** The models were fit to groups of three contiguous years of Mod Waters data at different spatial scale.
(TIFF)

Table S1 **Bias of contiguous models when predicting different spatial scales.** Bins were created to examine bias at varying days since dry (0-250, 251-500, 501-750, and >1000). Numbers represent the number of bins that had a bias value > 5. In all cases where there were spikes in bias values, these occurred in bins that had the largest DSD.
(TIFF)

Table S2 **Bias of non-contiguous models when predicting different spatial scales.** Bins were created to examine bias at varying days since dry (0-250, 251-500, 501-750, and >1000). Numbers represent the number of bins that had a bias value > 5. As in table S1, in all cases where there were spikes in bias values, these occurred in bins that had the largest DSD.
(TIFF)
