# Supplementary figures and images for: Space-for-Time Substitution Works in Everglades Ecological Forecasting Models
Source: PLoS One. 2013 Nov 21;8(11):e81025. doi: 10.1371/journal.pone.0081025 (PMC3836997; doi:10.1371/journal.pone.0081025)

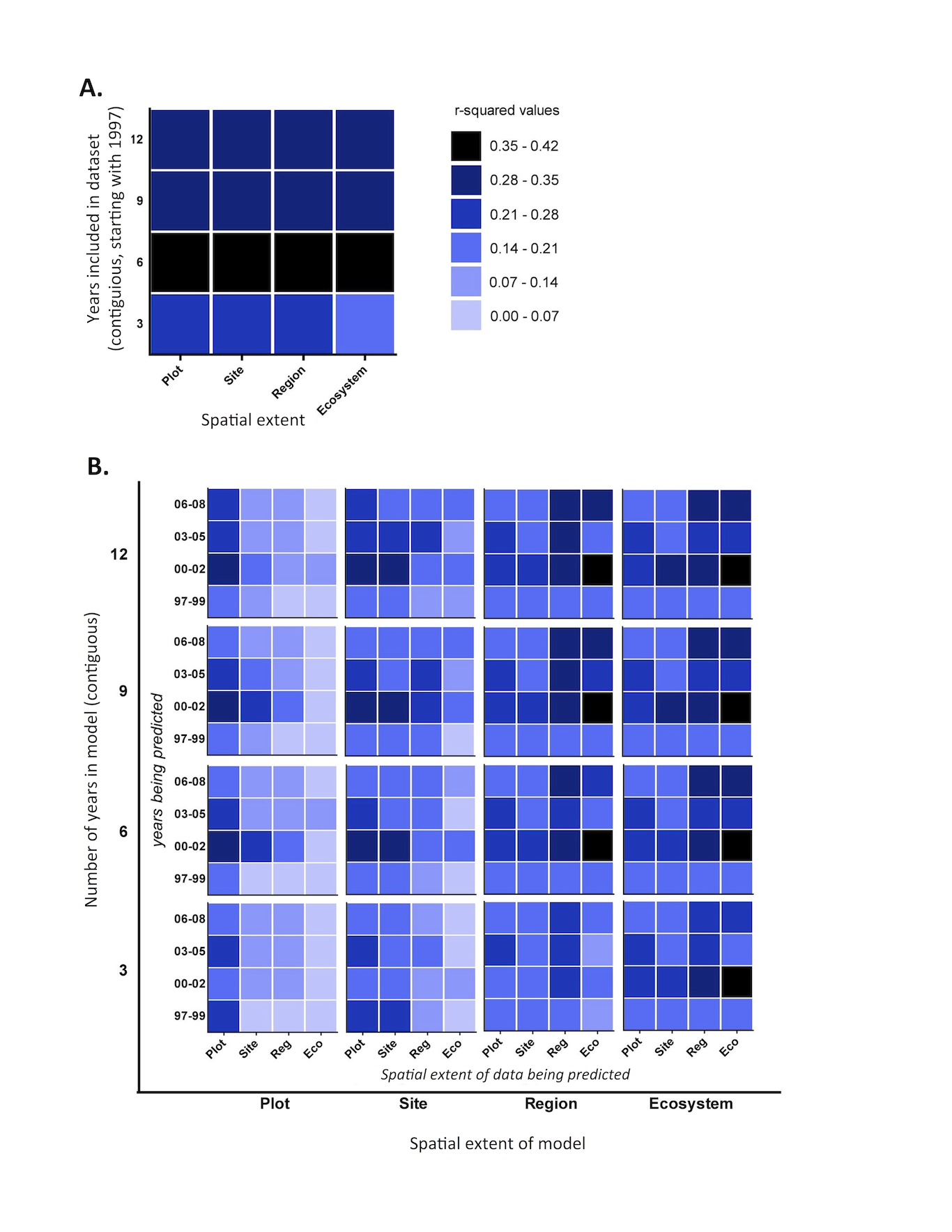

Supplement: Figure S1 — Heat map of contiguous year model fits. A) “Heat map” representation of fit for models created from a) contiguous years from the Mod Waters Data and b) The models from (a) fit to all combinations of groups of three contiguous years and spatial scales. (TIFF) [file pone.0081025.s001.tiff]

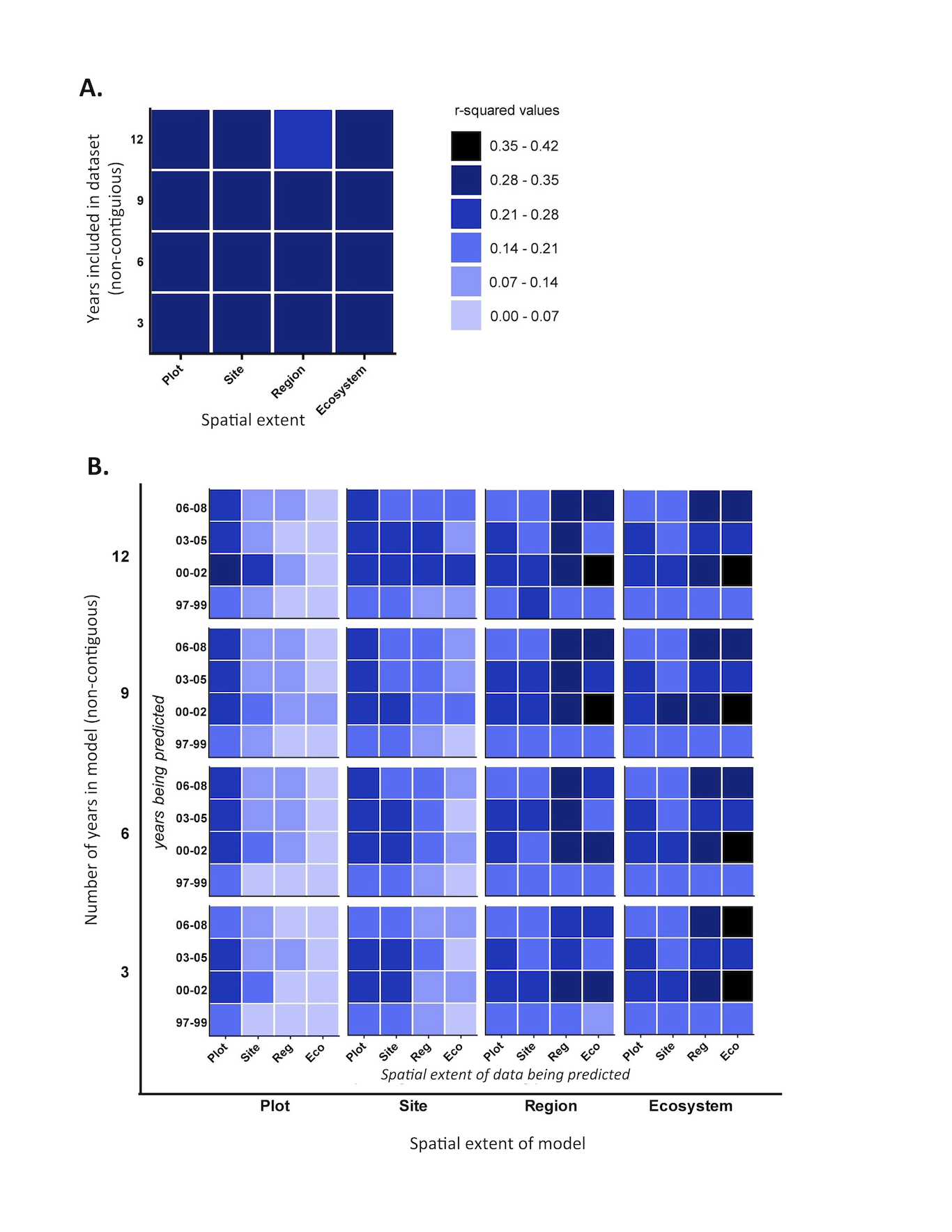

Supplement: Figure S2 — Heat map of non-contiguous year model fits. A) “Heat map” representation of fit for models created from a) non-contiguous years from the Mod Waters Data and b) The models from (a) fit to all combinations of groups of three contiguous years and spatial scales. (TIFF) [file pone.0081025.s002.tiff]

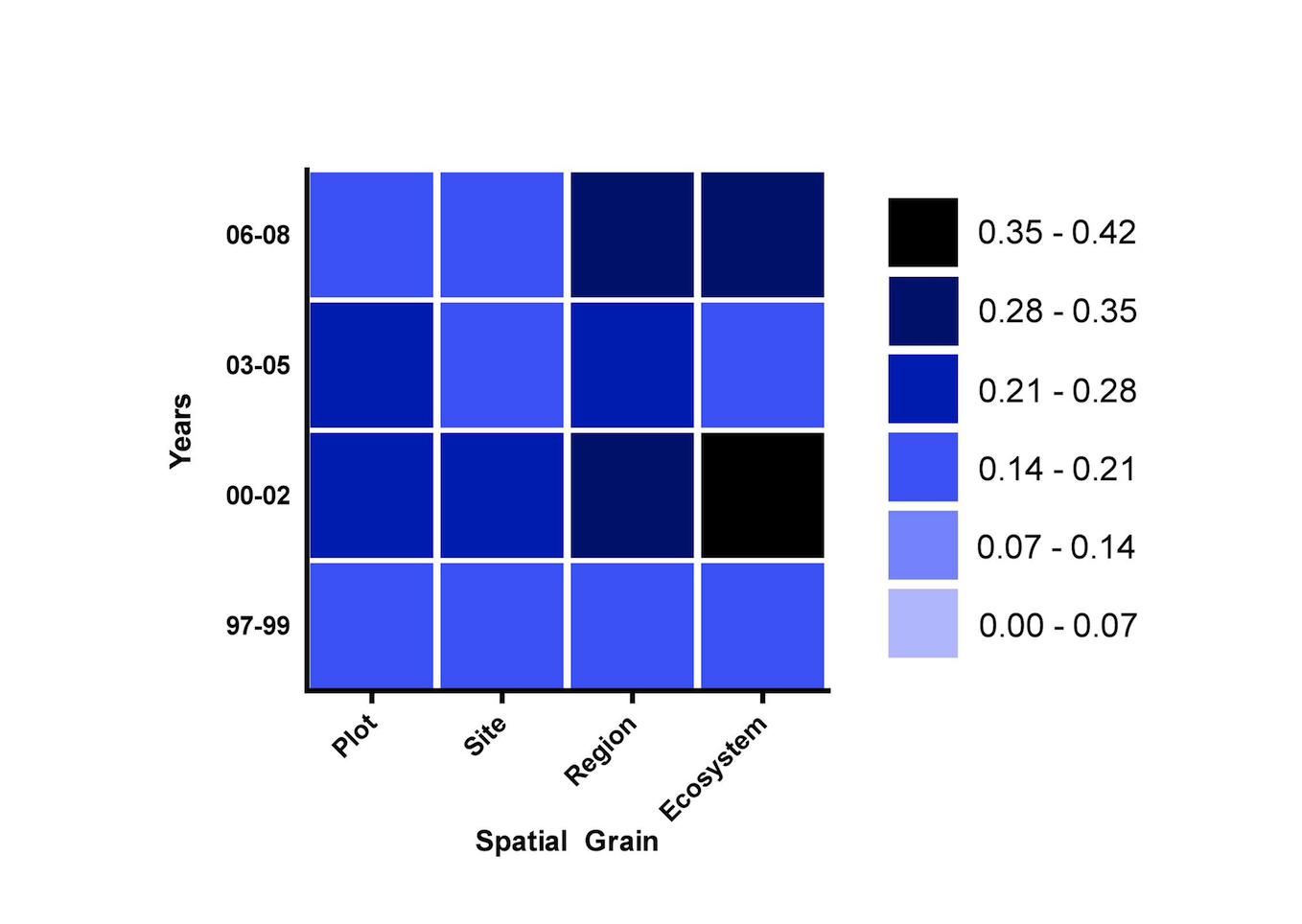

Supplement: Figure S4 — Mean r2 values when models from CERP data predict Mod Waters data. The models were fit to groups of three contiguous years of Mod Waters data at different spatial scale. (TIFF) [file pone.0081025.s004.tiff]

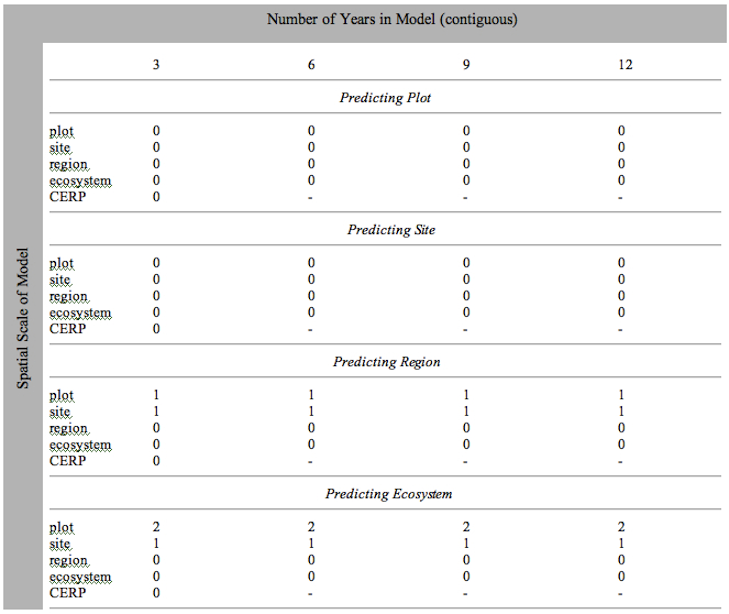

Supplement: Table S1 — Bias of contiguous models when predicting different spatial scales. Bins were created to examine bias at varying days since dry (0-250, 251-500, 501-750, and >1000). Numbers represent the number of bins that had a bias value > 5. In all cases where there were spikes in bias values, these occurred in bins that had the largest DSD. (TIFF) [file pone.0081025.s005.tiff]

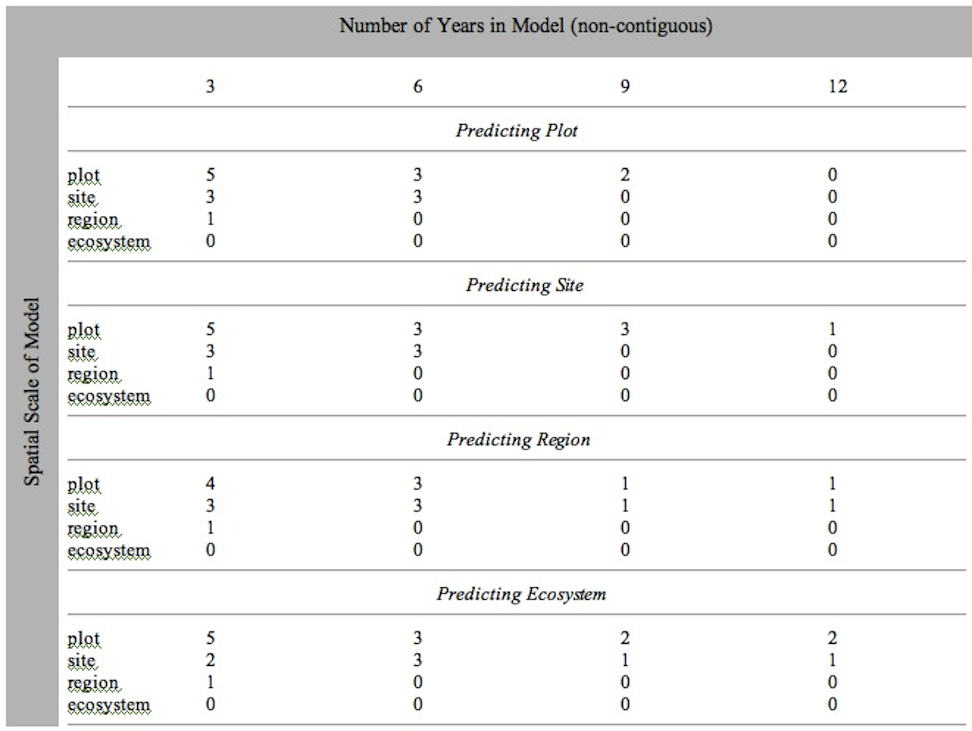

Supplement: Table S2 — Bias of non-contiguous models when predicting different spatial scales. Bins were created to examine bias at varying days since dry (0-250, 251-500, 501-750, and >1000). Numbers represent the number of bins that had a bias value > 5. As in table S1, in all cases where there were spikes in bias values, these occurred in bins that had the largest DSD. (TIFF) [file pone.0081025.s006.tiff]
